# MOCVD Growth of κ‑Ga_2_O_3_ on Al-Rich Al_*x*_Ga_1–*x*_N Templates: Phase Diagram and Microstructural Evolution

**DOI:** 10.1021/acs.cgd.6c00267

**Published:** 2026-04-29

**Authors:** Khai D. Ngo, Usman Ul Muazzam, Arpit Nandi, Sai K. Anandan, Yidi Yin, David Cherns, Menno J. Kappers, Rachel A. Oliver, Matthew D. Smith, Martin Kuball

**Affiliations:** † HH Wills Physics Laboratory, 1980 University of Bristol, Bristol BS8 1TL, U. K.; ‡ Department of Materials Science and Metallurgy, 2152 University of Cambridge, Cambridge CB3 0FS, U. K.

## Abstract

Orthorhombic κ-phase Ga_2_O_3_ is a metastable, ferroelectric polymorph of Ga_2_O_3_ with a large spontaneous polarization, offering a pathway to polarization-induced high-mobility 2D electron gases via κ-Ga_2_O_3_/Al_*x*_Ga_1–*x*_N heterostructures. Here, we map the metal–organic chemical vapor deposition (MOCVD) growth window of κ-Ga_2_O_3_ on Al-rich Al_*x*_Ga_1–*x*_N-on-sapphire template layers (*x* = 0.5 and 0.75) by systematically varying the gallium and oxygen precursor flow rates and benchmark these results against cogrown films on c-plane sapphire. On Al_0.5_Ga_0.5_N, κ-Ga_2_O_3_ can be grown over a wide range of conditions extending to low growth rates and high VI/III regions, whereas on sapphire substrates the κ-phase favorable window is restricted to the high growth rate and low VI/III regime only. Microstructural and phase evolution analyses by X-ray diffraction (XRD) and transmission electron microscopy (TEM) of the κ-Ga_2_O_3_ films grown on Al_0.5_Ga_0.5_N confirms that growth initiates as a phase-pure monoclinic β-Ga_2_O_3_ layer; κ-Ga_2_O_3_ nucleation starts between 20 and 45 nm of layer thickness and becomes the only phase growing around ∼100–250 nm thick, resulting in a phase-pure κ-Ga_2_O_3_ top surface. A similar progression was observed for growth on *x* = 0.75% Al_*x*_Ga_1–*x*_N template layers.

## Graphical abstract

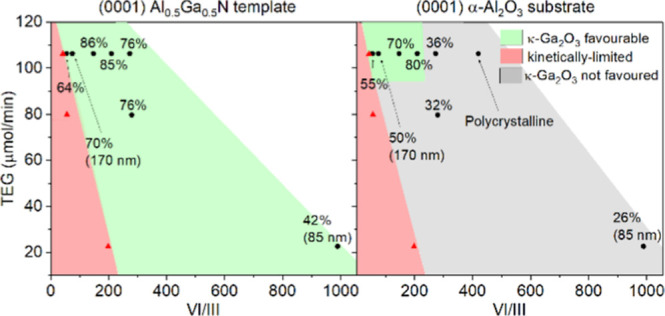

## 1 Introduction

Owing to its ultrawide bandgap and high critical electric field (up to 8 MV/cm), Ga_2_O_3_ is a promising material for solar blind UV–C photodetectors,^1,2^ and electronic devices in high voltage, high power applications (e.g., power grids, high-voltage transmission lines, electric vehicles)^3,4^ and in harsh environments operation with high radiation exposure.^5,6^ Among the many reported polymorphs (i.e., crystal phases) of Ga_2_O_3_, monoclinic β-Ga_2_O_3_ is the most thermodynamically stable phase and can be grown from the melt. Indeed, the availability of bulk-grown native substrates enabled the rapid advancement in β-Ga_2_O_3_ homoepitaxy^4,7^ and power electronic device demonstrations benefiting from the large bandgap of Ga_2_O_3_, ranging from 4.8 to 5.3 eV, depending on the polymorph.^8–14^ In recent years, metastable phases of Ga_2_O_3_ are gaining increasing attention owing to properties beneficial to devices that the β-phase lacks. Orthorhombic κ-Ga_2_O_3_ (space group *Pna*2_1_) is particular interesting for being a ferroelectric with a large predicted spontaneous polarization (P_SP_) of 23–26 μC cm^–2^.^15–18^ Due to the polarization charge, unlike monoclinic β-Ga_2_O_3_, no modulation doping would be required to form a high-mobility 2D electron gas (2DEG) channel at κ-(AlGa)_2_O_3_/κ-Ga_2_O_3_ heterojunctions. The reduced impurity scattering in a 2DEG offers a pathway to high breakdown, low on-resistance Ga_2_O_3_-based high electron mobility transistors (HEMTs).^19^ However, the epitaxial alloying of κ-Ga_2_O_3_ with Al to form κ-(AlGa)_2_O_3_ is scarcely explored, and the few reports available have exclusively used pulsed laser deposition (PLD).^20,21^

Al_*x*_Ga_1–*x*_N is a well-studied family of polar semiconductors with tunable bandgaps between 3.4 and 6.1 eV. With wide bandgaps exceeding that of κ-Ga_2_O_3_ and potentially large conduction band offsets relative to κ-Ga_2_O_3_,^22^ Al-rich Al_*x*_Ga_1–*x*_N (*x* > 0.5) is an attractive material for the back-barrier layer in κ-Ga_2_O_3_/Al_*x*_Ga_1–*x*_N heterojunctions. Theoretical calculations predicted 2DEG sheet charge densities up to 10^13^ −10^14^ cm^–2^ at these heterointerfaces, which is approximately 10 times that of typical Al_*x*_Ga_1–*x*_N/GaN heterostructures.^23–25^ Furthermore, the large dielectric constant and intrinsic polarity of κ-Ga_2_O_3_ have motivated its exploration as a gate dielectric in AlGaN/GaN HEMTs, with the potential to deliver improved electrical characteristics compared to conventional amorphous Al_2_O_3_ dielectrics.^26^ Together, these attributes make direct κ-Ga_2_O_3_/Al_*x*_Ga_1–*x*_N integration a promising avenue for power and high-frequency electronic devices.

However, the epitaxy of κ-Ga_2_O_3_ is challenging due to its metastability. There is a high tendency for stable β-Ga_2_O_3_ inclusions to form, even at low growth temperatures (<700 °C), which could drastically reduce the net polarization compared to the P_SP_ ideal figure and diminish the chances of forming a 2DEG channel in a heterostructure. Various epitaxial techniques have been employed to stabilize κ-Ga_2_O_3_, including molecular beam epitaxy (MBE),^27^ pulsed laser deposition (PLD),^22,28,29^ mist chemical vapor deposition (mist-CVD),^30–35^ and halide vapor phase deposition (HVPE).^36–39^ Compared to these techniques, industry-preferred MOCVD is distinguished by its capability of depositing high-quality thin films with excellent repeatability and scalability. To grow κ-Ga_2_O_3_ by MOCVD, two types of oxygen precursors are often reported: pure oxygen gas,^40–42^ and deionized (DI) water.^43,44^ The use of DI water instead of oxygen gas (as in conventional systems) modifies the growth chemistry and kinetics significantly, resulting in different growth temperature windows: 550–700 °C for DI water,^43^ 500–540 °C for pure oxygen gas.^41^ So far, deposition is most often realized on c-plane sapphire due to its ease of accessibility, and the growth windows are relatively well-known. However, they are rather narrow when using oxygen gas as precursor, while DI water typically allows a somewhat broader range of admissible growth conditions. Nevertheless, because of the mismatch in crystal symmetry and lattice spacing, κ-Ga_2_O_3_ films on c-plane sapphire typically suffer from multiple defects such as rotational domains, antiphase boundaries, twin boundaries, and stacking faults.^41,45–47^ Growth often begins as a thin β–Ga_2_O_3_ transition layer at the film/substrate interface, consistently observed across different epitaxial techniques: MBE,^27^ mist-CVD,^31^ and MOCVD (both types of oxygen precursor).^41,47^

Compared to the growth on c-plane sapphire, the growth of κ-Ga_2_O_3_ on Al_*x*_Ga_1–*x*_N by MOCVD is relatively underexplored. While the effects of growth temperature and precursor supersaturation on the phase competition and crystal quality have been characterized,^47,48^ the effects of VI/III ratio are less well studied. In terms of growth substrates, most MOCVD growth studies reported using GaN substrates,^23,43,44^ and only two works reported growing on AlN to date.^48,49^ In principle, the substrate Al composition determines factors such as epilayer/substrate strain, interfacial energy, surface energies and so forth, which could in turn influence the phase competition between β- and κ-Ga_2_O_3_. For growth on GaN, recent high-resolution transmission microscopy (HR-TEM) analysis confirmed a thin β–Ga_2_O_3_ transition layer between κ-Ga_2_O_3_ and GaN substrate.^47^ Models based on classical nucleation theory suggest that the growth mechanism on GaN and c-plane sapphire is similar: nucleation always begins as a thin β–Ga_2_O_3_ transition layer, on which κ-Ga_2_O_3_ subsequently grows.^47^ In contrast, observations on AlN vary. TEM studies of the film/substrate interface reported either nucleation of a mixture of β- & κ-Ga_2_O_3_ grains (DI water precursor),^49^ or amorphous Ga_2_O_3_ (oxygen precursor).^48^ Given the discrepancy in the reported film–substrate interface structures and oxygen precursor types, the growth mechanism of κ-Ga_2_O_3_ on Al-rich Al_*x*_Ga_1–*x*_N has not yet been fully established. Furthermore, it is also worth noting that only thick layers (250–1000 nm) were analyzed. Since κ→ β phase transformation is possible as the epilayer grows thicker,^50^ it is important to also characterize the epilayer during early stages of growth (<50 nm) to better understand the growth mechanism.

Therefore, in this study, the growth windows of κ-Ga_2_O_3_ on Al_*x*_Ga_1–*x*_N-on-sapphire template layers (*x* = 0.50 and 0.75) were mapped by systematically varying the flow rates of gallium and oxygen precursor gases. The microstructure and phase composition of the films at various stages of growth (from 20 to 1000 nm) on Al_*x*_Ga_1–*x*_N were carefully analyzed by XRD and TEM. Our results provide valuable guidance on the growth parameter space and template selection for κ-Ga_2_O_3_ growth by MOCVD.

## 2 Experimental Details

Ga_2_O_3_ films were grown using an Agnitron Agilis 100 MOCVD reactor in close-injection showerhead (CIS) configuration. Ga_2_O_3_ growth was performed on 10 × 10 mm^2^ templates cleaned ex-situ by solvents prior to epitaxy, on three different substrates for comparison: c-plane (0001) wurtzite Al_*x*_Ga_1–*x*_N-on-sapphire templates with compositions *x* = 0.50 and 0.75, and c-plane sapphire (all samples coloaded for simultaneous deposition). All the Al_*x*_Ga_1–*x*_N-on-sapphire templates employed a AlN nucleation layer and a 1000 nm thick AlN buffer layer. For the template with *x* = 0.75, 600 nm of Al_*x*_Ga_1–*x*_N was grown directly on the AlN buffer. For the template with *x* = 0.5, a 200 nm thick Al_*x*_Ga_1–*x*_N layer with *x* = 0.75 was first grown on the AlN buffer and a 600 nm Al_*x*_Ga_1–*x*_N layer with *x* = 0.5 was then grown on top of that.

Triethylgallium (TEGa) and high-purity O_2_ gas (99.9999%) were used as Ga and O precursors, with argon (Ar) carrier gas. The growth temperature *T*_gr_ and total chamber pressure p_T_ were fixed at 500 °C and 10 Torr, respectively, to facilitate the growth of κ-Ga_2_O_3_.^41,42^ To avoid the surface oxidation of the Al_*x*_Ga_1–*x*_N layers, oxygen gas was flowed simultaneously with TEGa during the deposition stage only i.e. the reactor chamber was under pure Ar environment during warm-up, temperature ramp, and cool-down stages. The combined effects of growth rate and VI/III ratio on Ga_2_O_3_ phase selection were investigated by varying the flow rate of TEGa between 85 and 400 sccm (corresponding to 22.6–106 μmol/min), and the flow rate of O_2_ between 100 and 500 sccm (4.46–22.3 mmol/min). The Ar carrier flow value was adjusted to ensure a constant total flow rate of 4000 sccm into the gas chamber. For this set of experiments, unless stated otherwise, the deposition time was fixed at 10 min. The microstructural evolution of the Ga_2_O_3_ films was subsequently investigated by fixing the growth condition and varying deposition time between 1 and 40 min.

After deposition, the Ga_2_O_3_ films were characterized by X-ray diffraction (XRD, Rigaku SmartLab Studio X-ray diffractometer equipped with a 3 kW Cu K_α_ source and 2-bounce Ge(220) monochromator) and tapping mode atomic force microscopy (AFM, Bruker Dimension Edge). Cross-sectional samples for transmission electron microscopy (TEM, FEI CM 200) and high-resolution transmission electron microscopy (HR-TEM, JEOL JEM-2100F) were prepared by focused ion beam milling (FIB, Thermo Fisher Helios 5 UX). The film thickness was measured by optical reflectometry (Filmetrics F20) using the refractive indices reported by Penman et al.^51^

## 3 Results and Discussion

### 3.1 Effect of Growth Rate and VI/III Ratio on the Phase Selection and Microstructure of Ga_2_O_3_ Films

First, the effects of VI/III ratio on Ga_2_O_3_ growth at higher growth rates were investigated by fixing the TEGa flow rate at 400 sccm (106 μmol/min). At this TEGa flow rate, the growth rate was found to be constant at 1.5 μm/h for VI/III ratios ≥150. Below a VI/III ratio of 150, the growth rate begins to decrease as the growth begins to enter the kinetically limited regime (see Figure S1 in Supporting Information). Thus, for the same growth time of 10 min, the resultant Ga_2_O_3_ films are 250 nm thick for samples for VI/III ≥150, but e.g. only 170 nm thick for VI/III ratios of 75. The deposition time was therefore increased by ∼10% for samples grown with VI/III = 55 to compensate for the reduction in growth rate. At VI/III = 42, we found that no sizable amount of deposition occurred.

Figure 1a shows the XRD 2θ-ω scans of Ga_2_O_3_ films grown on the Al_0.5_Ga_0.5_N templates for various VI/III ratios and constant TEGa flow of 106 μmol/min (1.5 μm/h growth rate). All patterns show two families of Ga_2_O_3_ peaks: the {002} κ-Ga_2_O_3_ reflections (“{002}­κ” in Figure 1) around 2θ = 19.1°, 38.8°, and 59.8°; and the {2̅01} β-Ga_2_O_3_ reflections (“ {2̅01}­β” in Figure 1) shifted a few tenths of degrees to the left of the {002}­κ peaks, at around 2θ = 18.9°, 38.3°, and 59.1°. Full XRD spectra including peaks from the substrate template can be found in Supporting Information (Figure S2). The detection of both families of peaks as shown in Figure 1a indicates the coexistence of monoclinic β- and orthorhombic κ-grains in the Ga_2_O_3_ film. The volume fraction of κ-grains in the film was estimated from XRD data using the following method. For thin film epitaxial samples, XRD intensities are approximately proportional to the structure factor squared |F_hkl_|^2^ and the volume of matter illuminated by the X-ray beam, V. Thus, the κ-phase fraction (KPF) of the film volume diffracted by X-ray was estimated by

1 $$\frac{V_{\kappa }}{V_{\kappa }+V_{\beta }}=\frac{1}{1+V_{\beta }/V_{\kappa }}∼\frac{1}{1+I_{\beta }{\left|F_{\kappa }\right|}^{2}/I_{\kappa }{\left|F_{\beta }\right|}^{2}}$$

where *V*_κ_,*V*_β_ are the diffracted volume of κ- and β-grains; *I*_β_, *I*_κ_ are the intensities (above the baseline) of the (4̅02)­β and (004)­κ peaks; and *F*_β_, *F*_κ_ are their respective structure factors.^52^

**Figure 1:**
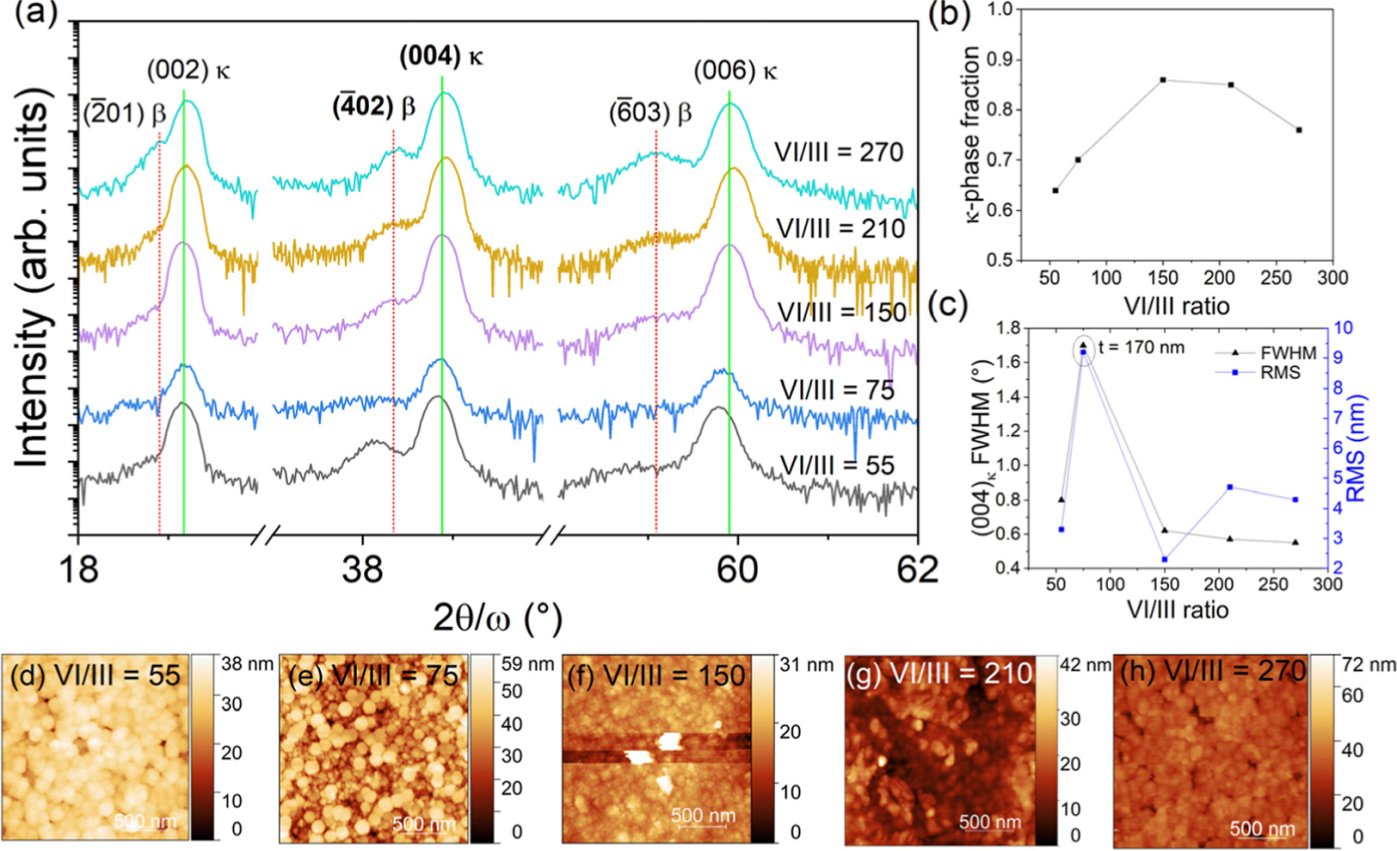
Structural characterization of Ga_2_O_3_ films grown on Al_0.5_Ga_0.5_N templates for VI/III ratios between 55 and 270 (constant TEGa = 106 μmol/min, *T*_gr_ = 500 °C, p_T_ = 10 Torr): (a) XRD 2θ-ω scans showing the {2̅01}­β and {002}­κ reflections; (b) κ-phase fraction (estimated using (4̅02)­β and (004)­κ peak intensities) against VI/III ratio; (c) XRC fwhm of the (004)­κ reflection and surface root-mean-square (RMS) roughness against VI/III ratio. AFM scans of a 2 × 2 μm^2^ area of the films for VI/III = (d) 55, (e) 75, (f) 150, (g) 210, (h) 270.

Using eq 1, the κ-phase fraction of the Ga_2_O_3_ films grown on Al_0.5_Ga_0.5_N are presented in Figure 1b. Overall, all Ga_2_O_3_ films grown on Al_0.5_Ga_0.5_N with high growth rates have KPF >50% and are therefore majority κ-Ga_2_O_3_. There is a maximum in the κ-phase fraction for VI/III ratios between 75 and 200, suggesting that κ-Ga_2_O_3_ growth is more favored in this window. The crystallinity of κ-phase material within the film was characterized by the full-width-half-maximum (fwhm) of the X-ray curve of (004)­κ peak. This was found to slightly improve as the VI/III ratio increases, with the lowest fwhm being 0.53° for VI/III ratios around 270. Note that the data point at a VI/III ration of 75 is an outlier since this film is thinner than the rest, as will become evident later. For all other samples that are 250 nm thick, AFM scans of Figure 1d,f-h show a surface morphology comprised of fully coalesced pseudohexagonal growth islands, consistent with that of κ-Ga_2_O_3_ reported by previous studies.^40,41,43^ These results, combined with the KPF analysis, indicate that the top surface is entirely κ-Ga_2_O_3_, which will be further supported by TEM discussed later.

To investigate the effects of VI/III ratio on the phase selection of Ga_2_O_3_ at lower growth rates, the TEGa flow rate was decreased to 300 and 85 sccm (80 and 22.6 μmol/min). For each TEGa flow rate, two oxygen flow rates were utilized: 100 and 500 sccm (4.46 and 22.3 mmol/min). The deposition time was kept constant at 10 min. XRD 2θ-ω scans (Figure S3 in Supporting Information) were performed for the resultant films, and the KPF was calculated. All results are summarized in Figure 2a, in which each data point represents a growth condition, and each number is the κ-phase fraction of the resultant Ga_2_O_3_ layer.

**Figure 2:**
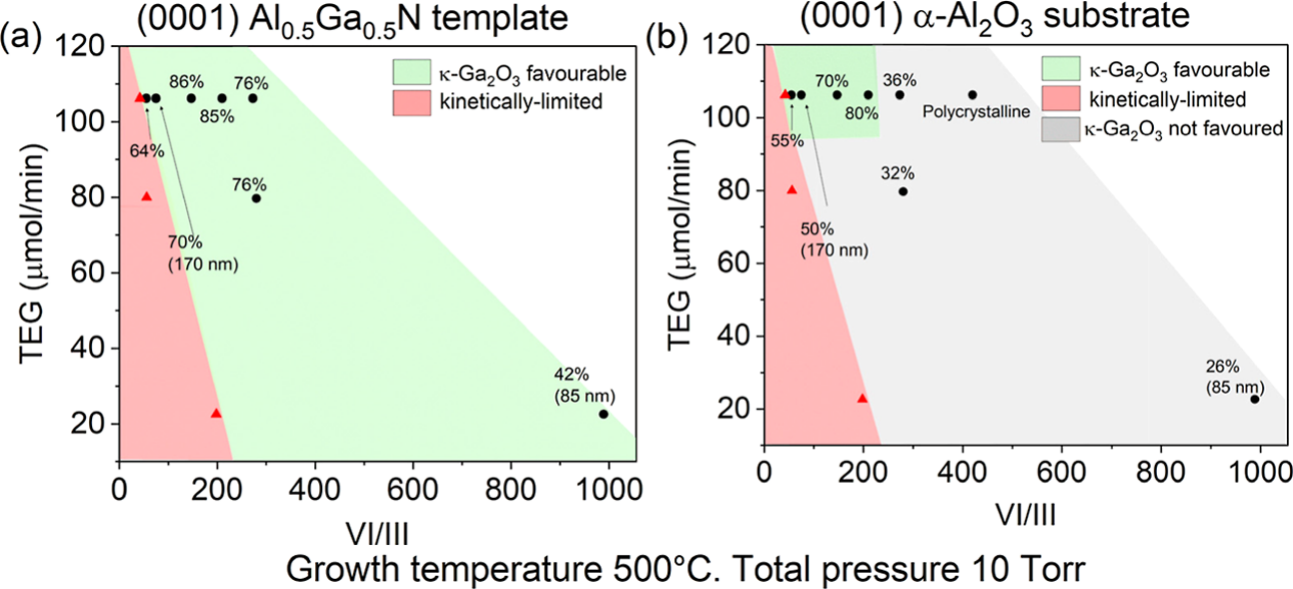
Growth phase diagram of Ga_2_O_3_ on (a) (0001) Al_0.5_Ga_0.5_N and (b) c-plane sapphire (*T*_gr_ = 500 °C, p_T_ = 10 Torr held constant). Regions shaded in red correspond to kinetically limited growth conditions, which would result in no epitaxial growth because of insufficient oxidation. Regions shaded in green correspond to κ-Ga_2_O_3_ favorable growth conditions, which would result in films with κ-phase fraction ≥50% at 200 nm thick. Gray regions indicate conditions where κ-Ga_2_O_3_is not favored. Note that the shaded regions serve only as guides to the eye, and boundaries between them are approximate. Unshaded regions (in white) correspond to unexplored conditions.

The conditions TEGa = 80 μmol/min, VI/III = 56 and TEGa = 22.6 μmol/min, VI/III = 200 did not result in sizable growth and therefore fall under the kinetically limited region to the left in Figure 2a. This is because gallium hydride (GaH_3_) created from TEGa pyrolysis reacts with O_2_ gas and may form either gallium suboxide (Ga_2_O) or gallium oxide (Ga_2_O_3_). Being a volatile molecule, Ga_2_O will typically desorb from the free surface and not contribute to film growth. Unless there is sufficient oxygen to further oxidize Ga_2_O into Ga_2_O_3_, the growth rate will be reduced to 0 (i.e., no growth) for very low VI/III ratios.^53^ We also observed that higher oxygen flow rates are required for epitaxy when at lower TEGa flows, in good agreement with theoretical models devised for β-Ga_2_O_3_ at 700 °C.^53^ On the other hand, based on the analysis of the majority κ-Ga_2_O_3_ films previously presented in Figure 1, the growth condition is deemed κ-Ga_2_O_3_ favorable if the resultant Ga_2_O_3_ film has KPF ≥50% for layers thicker than 200 nm. Otherwise, the condition is κ-Ga_2_O_3_ not favored. Of the low growth rate experiments, the condition TEGa = 80 μmol/min, VI/III = 280 resulted in a 200 nm thick film with KPF = 76% and thus is κ-Ga_2_O_3_ favorable. On the other hand, the condition TEGa = 22.6 μmol/min, VI/III = 990 resulted in an 85 nm thick film only due to the lower growth rate. Since the κ-phase fraction increases with layer thickness, which we will discuss later, a KPF of 42% at 85 nm is consistent with the growth of a majority κ-Ga_2_O_3_ film. Hence, the condition TEGa = 22.6 μmol/min, VI/III = 990 is also κ-Ga_2_O_3_ favorable.

For comparison, the same κ-phase fraction analysis was carried out for Ga_2_O_3_ films simultaneously grown on c-plane sapphire in Figure 2a, samples that were cogrown with the previously discussed samples in the same growth runs. The results are summarized in Figure 2b (XRD scans in Figure S4 in Supporting Information). Compared to the growth phase diagram on Al_0.5_Ga_0.5_N of Figure 2a, the kinetically limited region of Figure 2b is the same, since it is independent of the growth substrate. However, the κ-Ga_2_O_3_ favorable region in the phase diagram for growth on c-plane sapphire in Figure 2b is confined specifically to high growth rates and low VI/III ratio conditions only (top left corner of the phase diagram). Interestingly, if the VI/III ratio is too high, even at high growth rates, the resultant growth is polycrystalline (marked in Figure 2b). In contrast, the κ-Ga_2_O_3_ favorable window for Al_0.5_Ga_0.5_N templates extends further into lower growth rates (i.e low TEGa, down to ∼20 μmol/min) and even into higher VI/III ratio (up to ∼1000) regions. Selected experiments in Figure 2a were repeated on Al_0.75_Ga_0.25_N templates. Both XRD and AFM analysis (Figure S5 in Supporting Information) show that majority of κ-Ga_2_O_3_ films were also grown successfully on Al_0.75_Ga_0.25_N, with insignificant obvious difference in KPF compared to the layers grown on Al_0.5_Ga_0.5_N. These results indicate that phase selection is broadly similar across the Al-rich Al_*x*_Ga_1–*x*_N on sapphire templates examined.

### 3.2 Structural Evolution of Ga_2_O_3_ Film Grown under κ-Favorable Conditions on Al_*x*_Ga_1–*x*_N Templates

With growth conditions κ-phase having been identified, the microstructural evolution of the κ-Ga_2_O_3_ films on Al_0.5_Ga_0.5_N was investigated, focusing on samples with fixed TEGa flow rate at 106 μmol/min and VI/III ratio at 210. The growth time was varied between 1 and 40 min, producing layers between 20 and 1000 nm thick on Al_0.5_Ga_0.5_N templates. XRD 2θ-ω scans (see Figure 3a) reveal that in the initial stages of growth (after ∼1 min), the film nucleates as pure β-Ga_2_O_3_ on Al_0.5_Ga_0.5_N, as evidenced by the single peak around 2θ ∼38.0°–38.2°, attributed to (4̅02) β, in XRD. The surface at this point in time exhibits a morphology consisting of noncoalesced, predominantly β-phase islands (see Figure 3c), though there is a possibility that these islands nucleated on a very thin 2D β-Ga_2_O_3_ wetting layer. The average thickness of ∼20 nm was extrapolated from the average growth rate of 1.5 μm/h. In the next stage of growth (around 45 nm thick), the (004) κ peak first appears in Figure 3a XRD scans around 2θ ∼38.8°, and the top surface of the film is now completely covered by small spherical islands approximately 80–100 nm wide (Figure 3d). During this stage (between 20–100 nm thick), both phases are nucleating on the film simultaneously – as shown by the monotonic increase in intensity of both (004) κ and (4̅02) β peaks in the XRD scans, and so it is not possible to distinguish between κ- and β-islands in Figure 3d,e. As the layer continues to develop, however, the intensity of the (2̅01) β peak stays relatively constant, while the intensity of (004) κ experiences a sharp increase (by an order of magnitude) as thickness increases from 100 to 250 nm. Correspondingly, there is a sudden hike in the KPF, from ∼35% at 100 nm to ∼86% at 250 nm thick (Figure 3b). This means κ-Ga_2_O_3_ growth becomes more favored and begins to dominate over β-Ga_2_O_3_ after a certain layer thickness between 100 and 250 nm. Eventually, κ-Ga_2_O_3_ becomes the only phase growing when κ-grains fully coalesce over β-grains, confining the latter to the first layers only (which gives an almost constant shoulder peak to the left of {002}­κ peaks in XRD 2θ-ω). Partial κ-Ga_2_O_3_ coalescence can be observed in the AFM scan of the Ga_2_O_3_ film at 100 nm (Figure 3e) and 170 nm thick (Figure 1e, grown under a different condition). After full coalescence, the final surface of the film is pure κ-Ga_2_O_3_, with the morphology of Figure 3g,h. Correspondingly, the KPF approaches 100% as the film continues to grow thicker (Figure 3b). On Al_0.75_Ga_0.25_N, the time-dependent growths were repeated, and the same trends in KPF and surface morphology were observed (Figure S6 in Supporting Information), indicating that the same growth mechanism takes place on Al_*x*_Ga_1–*x*_N samples with both *x* = 0.50 and 0.75.

**Figure 3:**
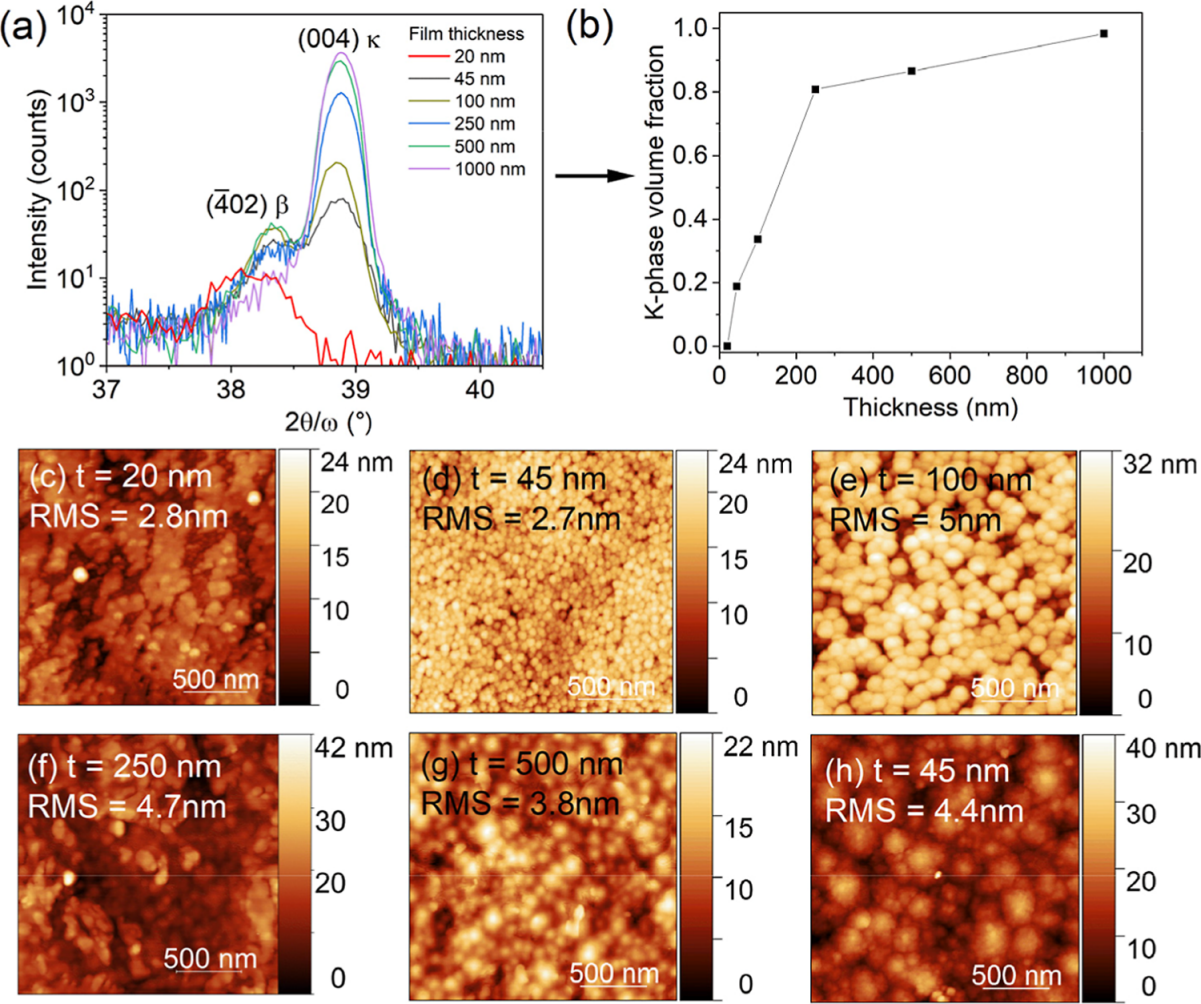
(a) XRD 2θ-ω scans, (b) κ-phase fraction, (c–h) AFM scans of Ga_2_O_3_ films 20–1000 nm thick grown on Al_0.5_Ga_0.5_N templates, under identical growth conditions (TEG = 106 μmol/min, VI/III = 210, *T*_gr_ = 500 °C, p_T_ = 10 Torr).

Cross-sectional TEM analysis of the 250 nm thick film (KPF ∼86%) grown on Al_0.5_Ga_0.5_N confirms the proposed growth mode. The bright-field (BF) image (Figure 4a) shows columnar growth of Ga_2_O_3_ on Al_0.5_Ga_0.5_N. Selected area electron diffraction (SAED) patterns from surface regions the Ga_2_O_3_ film (Figure 4b) and the Al_0.5_Ga_0.5_N template (Figure 4c) are consistent with the <010>/⟨310⟩ zone axis (ZA) of κ-Ga_2_O_3_ and the <112̅0> ZA of Al_0.5_Ga_0.5_N, respectively. No other phases were detected, indicating that top surface of the film is pure κ-Ga_2_O_3_, in agreement with AFM and XRD. Note that due to double diffraction, forbidden reflections such as (0001) Al_0.5_Ga_0.5_N and (001) κ-Ga_2_O_3_ are visible. In addition, in Figure 4b, rows of extra diffraction spots between {00l} and {20l} reflections are attributed to streaking effect arising from {110}-twinned nanocrystallites;^46^ the faint amorphous ring comes from gold sputtered on the film to prevent charging during FIB lamella preparation. At the film/Al_0.5_Ga_0.5_N interfacial regions, the high magnification HR-TEM image (Figure 5) reveals the presence of a transition layer comprised of β-Ga_2_O_3_ islands (or islands +2D wetting layer) nucleating during the first stages of deposition between κ-Ga_2_O_3_ and the Al_0.5_Ga_0.5_N template layer. κ-Ga_2_O_3_ appears to grow on top of the sidewalls of these initial β-Ga_2_O_3_ islands (approximated by dotted yellow lines in Figure 5a). The angles between the β-Ga_2_O_3_ sidewalls and the interface are typically in the 47–55° range, which might suggest that the sidewalls are of {001} or {100}-type, consistent with the Wulff shape of β-Ga_2_O_3_ islands on substrates with hexagonal symmetry like (0001) Al_*x*_Ga_1–*x*_N.^54^ Other regions along the film/interface show a phase distribution consistent with Figure 5 (Figure S7 in Supporting Information), indicating that the transition layer is continuous throughout the sample. Because thinner films contain a larger contribution of mixed phase interfacial regions, they are expected to exhibit worse crystal quality compared to thicker films, as previously noted in Section 3.1.

**Figure 4:**
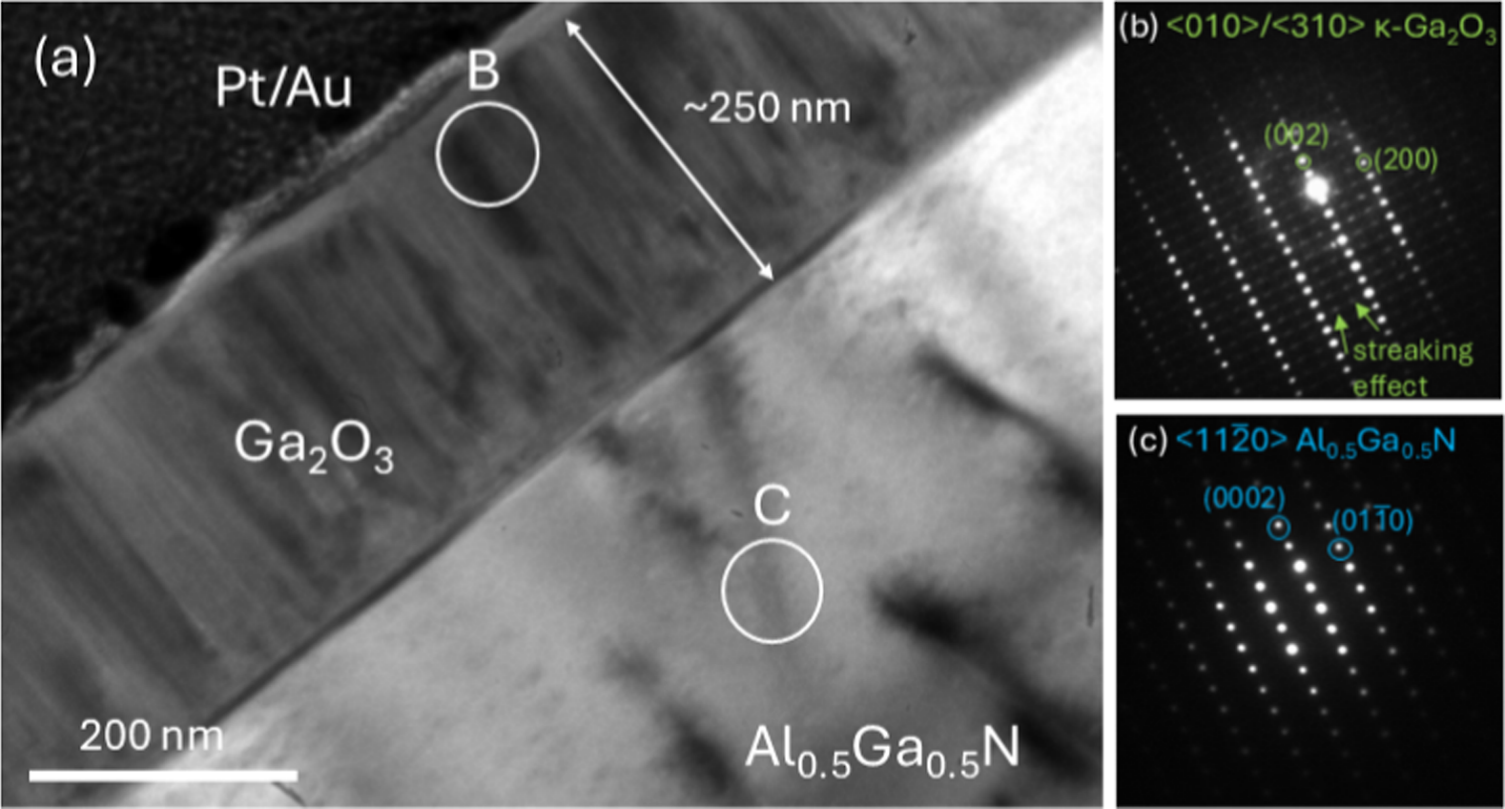
(a) Bright-field TEM image of the 250 nm κ-Ga_2_O_3_ film (KPF ∼86%) on Al_0.5_Ga_0.5_N under the conditions TEG = 106 μmol/min, VI/III = 210, *T*_gr_ = 500 °C, p_T_ = 10 Torr. Selected area electron diffraction (SAED) patterns of: (b) region B in (a) showing the <010>/⟨310⟩ zone axis (ZA) of κ-Ga_2_O_3_, (c) region C in (a) showing the <112̅0> ZA of Al_0.5_Ga_0.5_N.

**Figure 5:**
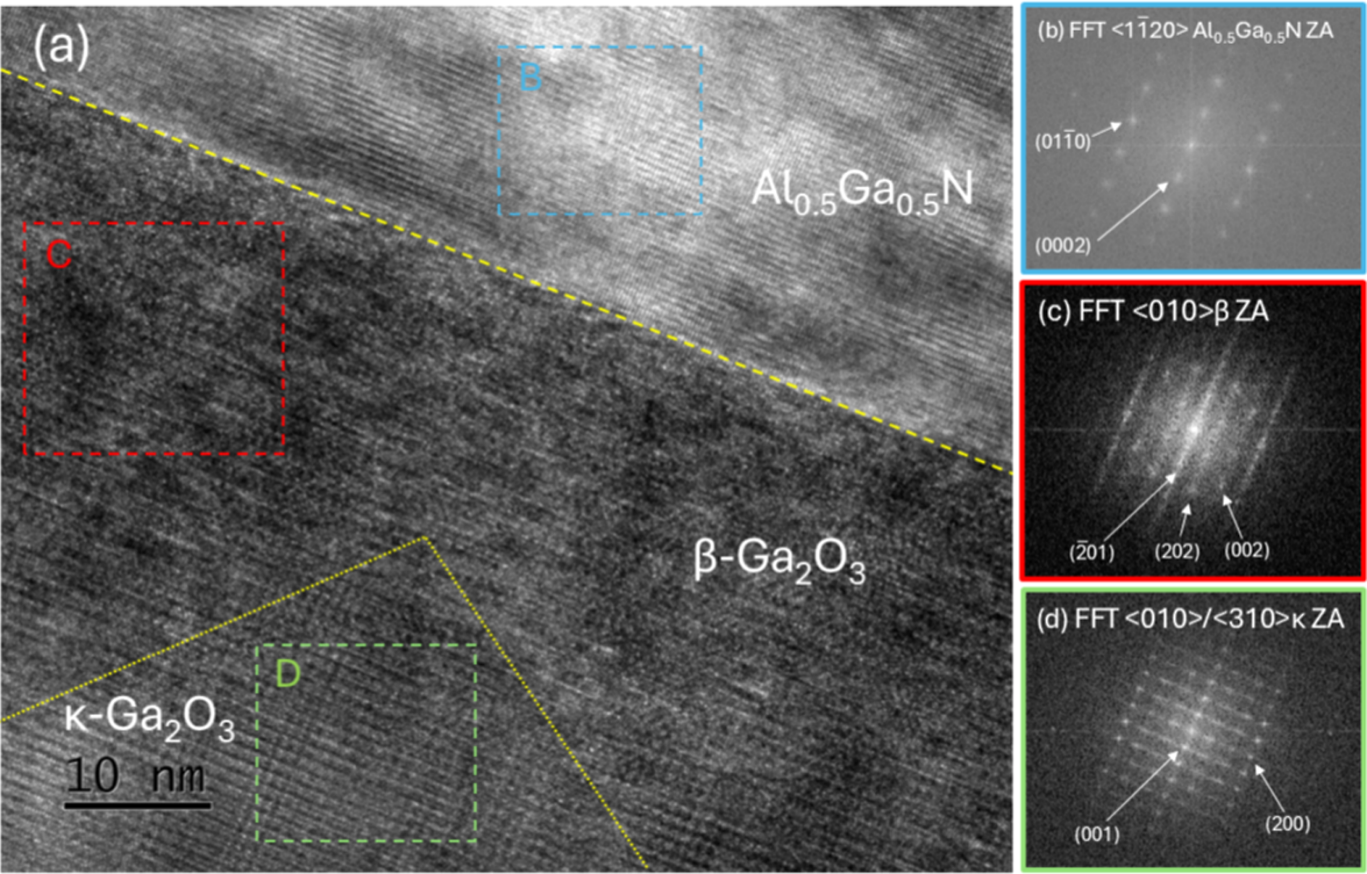
(a) High magnification HR-TEM image of a region near the film/Al_0.5_Ga_0.5_N interface of the 250 nm κ-Ga_2_O_3_ film (KPF ∼86%) on Al_0.5_Ga_0.5_N templates, grown using the conditions: TEG = 106 μmol/min, VI/III = 210, *T*_gr_ = 500 °C, p_T_ = 10 Torr. Dashed yellow line marks the film/Al_0.5_Ga_0.5_N interface, and dotted yellow lines mark the approximate boundaries between κ- and β-grains. Fast Fourier transforms (FFTs) of: (b) Region B (dashed blue square) showing the diffraction pattern (DP) of the <112̅0> ZA of Al_0.5_Ga_0.5_N, (c) Region C (dashed red square) showing the DP of the <010> ZA of β-Ga_2_O_3_, and (d) Region D (dashed green square) showing the DP of the <010>/⟨310⟩ ZA of κ-Ga_2_O_3_.

Our results so far indicate that, in MOCVD, κ-Ga_2_O_3_ grows on Al-rich Al_*x*_Ga_1–*x*_N through a β–Ga_2_O_3_ transition layer. This observation is in good agreement with reported growth on GaN,^47^ in which β-Ga_2_O_3_ was found to always nucleate first, and can be explained using the same model based on classical nucleation theory. In this model, the competition between β- and κ-Ga_2_O_3_ is determined by the Gibbs free energy Δ*G* gain of nucleating a growth islands, and the phase which has the greatest net energy gain will nucleate. Without an exact treatment of the island geometry, under simplified terms, Δ*G* is a sum of the condensation energy gain Δ*G*_V_, the net surface energy cost Δγ, the interface energy cost γ_int_, and the strain energy cost Δ*E*_s_. Out of these contributions, the condensation energy Δ*G*_V_ gain is greater for the stable phase β-Ga_2_O_3_, and the net surface energy cost Δγ is lower to form β-Ga_2_O_3_ island facets,^47,55,56^ according to DFT calculations. Through the selection of a suitable substrate, the interface energy γ_int_ and strain energy Δ*E*_s_ are controllable, with the strain energy generally being a critical factor. If the in-plane lattice mismatch of β-Ga_2_O_3_/substrate is disproportionately greater than that of κ-Ga_2_O_3_/substrate, the elastic energy cost Δ*E*_s_ to nucleate β-Ga_2_O_3_ could become prohibitively high, and κ-Ga_2_O_3_ is favored. Here, in the κ-Ga_2_O_3_ & β-Ga_2_O_3_ on Al_*x*_Ga_1–*x*_N system, the in-plane epitaxial relationship is <010>/⟨310⟩κ || < 010>β || < 112̅0> Al_*x*_Ga_1–*x*_N (confirmed by TEM in Figures 4–5). By domain matching epitaxy,^57^ the in-plane mismatch of (060)/{330} κ versus (112̅0) Al_*x*_Ga_1–*x*_N decreases from ∼9.1% to ∼4.3% as x increases from 0 to 1. Therefore, in principle, this trend suggests that κ-Ga_2_O_3_ formation may become more favorable on Al-rich Al_*x*_Ga_1–*x*_N compared to GaN—the latter of which exhibits the largest mismatch i.e. greatest strain energy cost of forming κ-Ga_2_O_3_ islands. However, at the same time, the in-plane lattice mismatch of (020) β versus (112̅0) Al_*x*_Ga_1–*x*_N also decreases with Al content (∼4% to ∼2% for *x* = 0 to *x* = 1) and remains consistently lower than the κ-Ga_2_O_3_/Al_*x*_Ga_1–*x*_N mismatch (∼9.1% to ∼4.3% for *x* = 0 to *x* = 1), across the full composition range. Hence, while lattice parameter considerations suggest improved κ-Ga_2_O_3_ favorability toward Al-rich Al_*x*_Ga_1–*x*_N (owing to lower elastic energy cost Δ*E*_s_), they also imply a parallel improved favorability for β-Ga_2_O_3_ nucleation. Taken together, these factors are consistent with our experimental observation that MOCVD growth of κ-Ga_2_O_3_ on Al-rich Al_*x*_Ga_1–*x*_N template layers always initiates through a β–Ga_2_O_3_ transition layer. This behavior shows little variation with the nominal Al content of the Al_*x*_Ga_1–*x*_N template layers, though it may also in part reflect that both templates were grown on sapphire and may therefore have similar lattice constants. Notwithstanding, in effect, since the κ-Ga_2_O_3_ film is always grown on the β–Ga_2_O_3_ transition layer, and not on the Al_*x*_Ga_1–*x*_N template layer, the resultant films appear similar in XRD and AFM for both *x* = 0.50 or 0.75 templates (compare Figures 3 and S6). The invariable formation of the β–Ga_2_O_3_ transition layer is important, since it implies that phase-pure κ-Ga_2_O_3_ on Al_*x*_Ga_1–*x*_N epitaxy by MOCVD will require suitable extra gases during growth to directly lower the formation energy κ-islands like HCl, TESn, or TMSn.^27,31,58^

## 4 Conclusions

We show that κ-Ga_2_O_3_ growth on Al-rich Al_0.5_Ga_0.5_N template layers by MOCVD is favored across a broad range of growth rates and VI/III ratios, unlike on c-plane sapphire where the κ-favorable window is specifically constrained to high growth rate and low VI/III ratios only. Analysis of the structural evolution of the Ga_2_O_3_ films on Al_0.5_Ga_0.5_N template layers under κ-Ga_2_O_3_ favorable conditions reveals three main stages. In the first stage, the β–Ga_2_O_3_ transition layer invariably forms, then, κ-Ga_2_O_3_ starts nucleating on the β–Ga_2_O_3_ transition layer at some point between 20 and 45 nm of layer thickness. This marks the start of the stage, during which grains of both polymorphs grow simultaneously. Eventually, growth enters the third stage when κ-Ga_2_O_3_ grains have coalesced and completely covered over β-grains, blocking the latter’s growth. This was seen to occur between 100 and 250 nm of layer thickness. Only κ-Ga_2_O_3_ grows in this stage, resulting in a phase-pure κ-Ga_2_O_3_ top surface. Overall, it appears that this growth mode on Al-rich Al_*x*_Ga_1–*x*_N templates is largely consistent with that reported for GaN at the low Al-composition limit.

## Supplementary Material



## Notes

Growth rate versus V/III ratio data; additional XRD 2θ-ω scans of Ga_2_O_3_ films grown on Al_*x*_Ga_1–*x*_N-on-sapphire templates for x = 0.5, x = 0.75, and on sapphire substrates; AFM scans of Ga_2_O_3_ films grown on Al_*x*_Ga_1–*x*_N (x = 0.75) templates; additional HR-TEM scans of the 250 nm κ-Ga_2_O_3_ film on Al_0.5_Ga_0.5_N templates (PDF)
