# Supplementary material for: MOCVD Growth of κ‑Ga2O3 on Al-Rich Al x Ga1–x N Templates: Phase Diagram and Microstructural Evolution
Source: Cryst Growth Des. 2026 Apr 29;26(10):4020–8. doi: 10.1021/acs.cgd.6c00267 (PMC13195645; doi:10.1021/acs.cgd.6c00267)
Supplement: Supplementary file 1 [file cg6c00267_si_001.pdf]

Supporting Information

MOCVD growth of κ-Ga_2O_3 on Al-rich $Al_xGa_{1-x}N$ templates: Phase diagram and microstructural evolution

Khai D. Ngo[1], Usman Ul Muzzam[1], Arpit Nandi[1], Sai Anandan[1], Yidi Yin[1], David Cherns[1], Yidi Yin[1], Menno Kappers[2], Rachel A. Oliver[2], Matthew D. Smith[1], and Martin Kuball[1,]**

[1]HH Wills Physics Laboratory, University of Bristol, Bristol BS8 1TL, United Kingdom

[2]Department of Materials Science and Metallurgy, University of Cambridge, Cambridge CB3 0FS, United Kingdom

E-mails: khai.ngo@bristol.ac.uk
Martin.Kuball@bristol.ac.uk

Section A: Effect of growth rate and VI/III ratio on the phase selection and microstructure of Ga_2O_3 films.

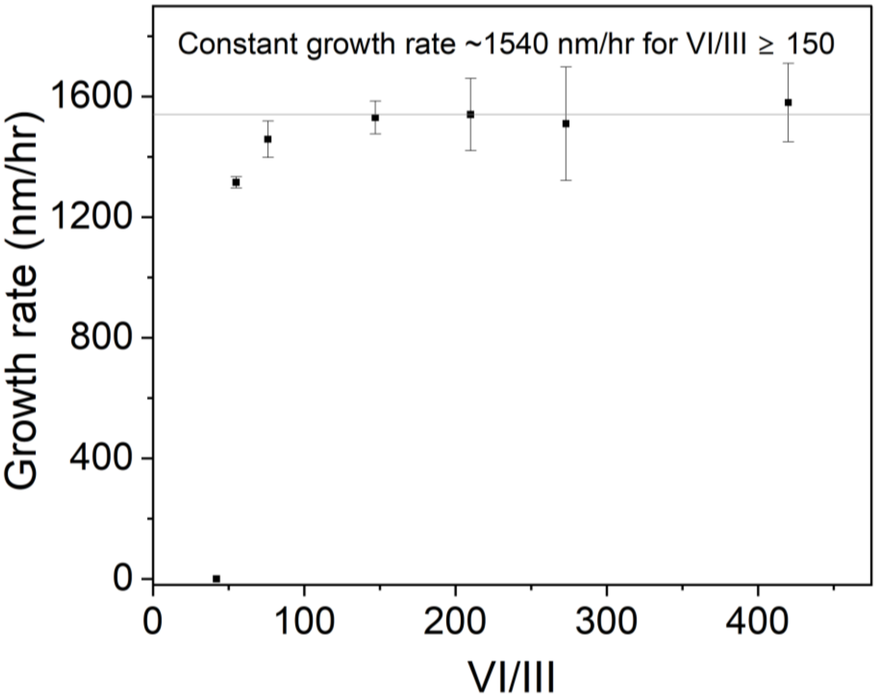

Figure S1: Growth rate (nm/hr) versus VI/III ratio for constant TEG flow rate = 400 sccm i.e. 106 μmol/min. The growth rate is constant at ~1540 nm/hr for VI/III ≥ 150, and drops to 0 (i.e. no growth) for very low values of VI/III (< 50).

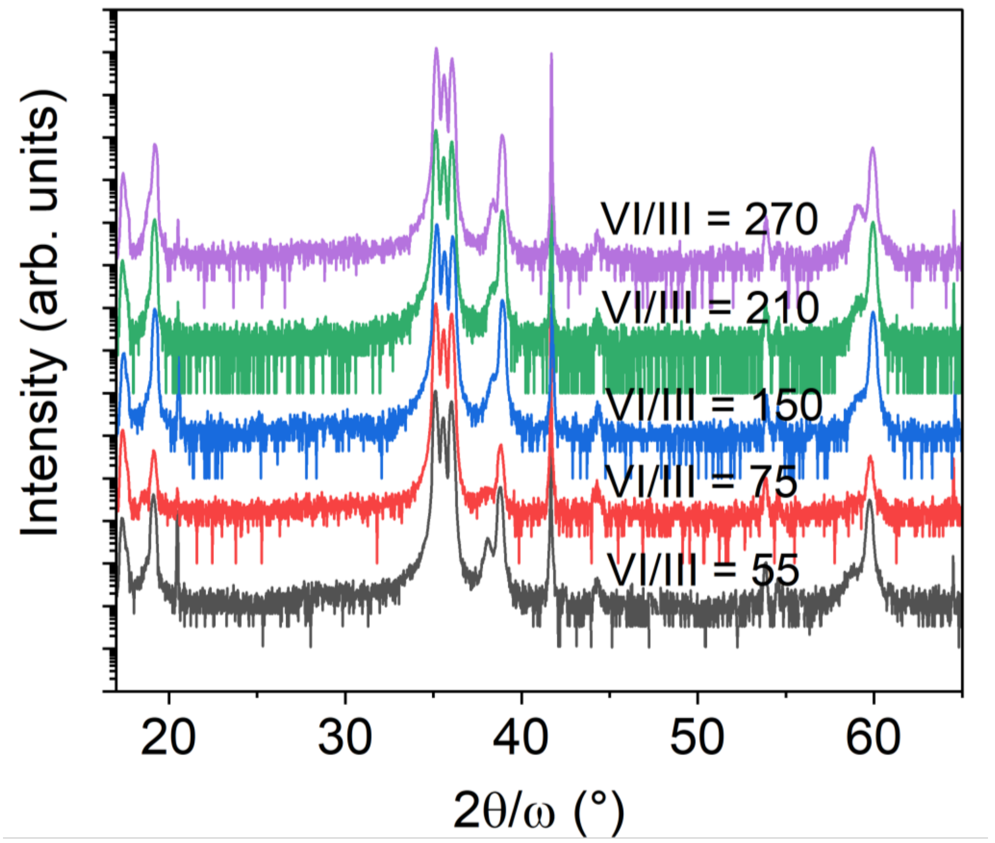

Figure S2: Full-range XRD 2θ-ω scans of Ga_2O_3 films grown on $Al_{0.5}Ga_{0.5}N$ templates for VI/III ratios between 55 – 270 (constant TEG = 106 μmol/min, T_{gr} = 500°C, p_T = 10 Torr). In all XRD patterns, the following peaks from the $Al_{0.5}Ga_{0.5}N$ template (on which deposition was performed) are present: (0006) α-Al_2O_3 peak from the sapphire substrate at 2θ = 41.7°; the three peaks between 35 - 36° correspond to (0002) $Al_{0.5}Ga_{0.5}N$, (0002) $Al_{0.75}Ga_{0.25}N$, and (0002) AlN (in that order). The $Al_{0.75}Ga_{0.25}N$ and AlN layers come from the strain-relief buffer stack of the template. Note that the peak at 2θ = 44.5° comes from the aluminium base plate, on which the sample was placed.

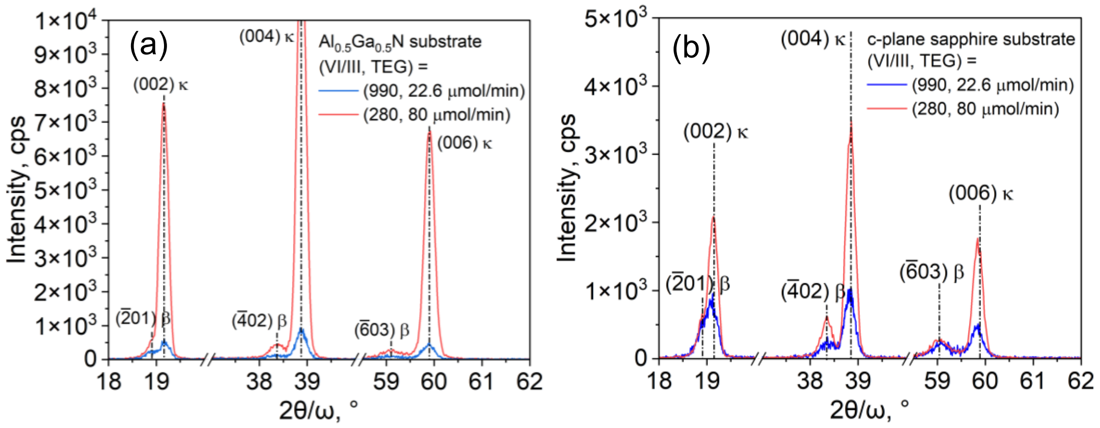

Figure S3: XRD 2θ-ω scans of Ga_2O_3 films grown on (a) $Al_{0.5}Ga_{0.5}N$ templates and (b) c-plane sapphire substrates for lower supersaturation conditions: (VI/III = 990, TEG = 22.6 μmol/min) and (VI/III = 280, TEG = 80 μmol/min).

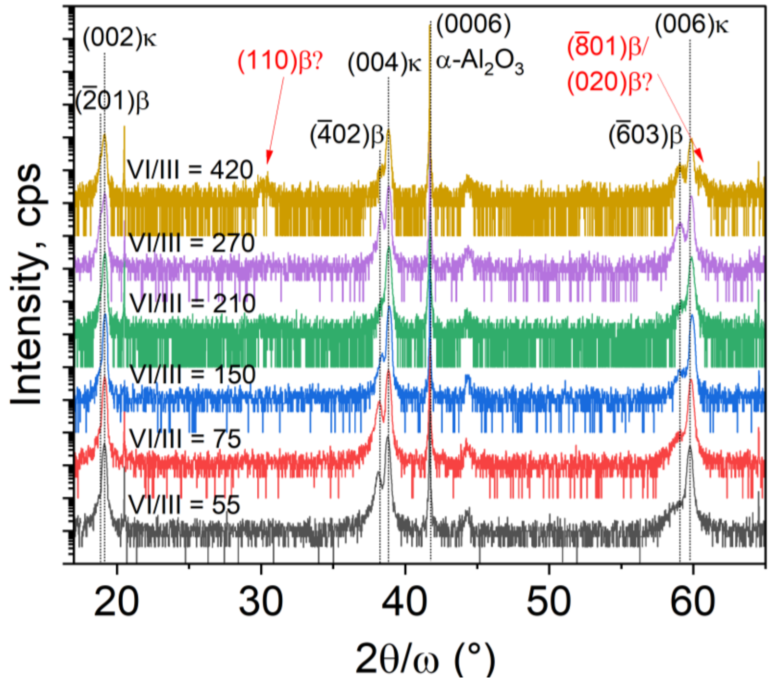

Figure S4: Full-range XRD 2θ-ω scans of Ga_2O_3 films grown on c-plane sapphire for VI/III ratios between 55 – 420 (constant TEG = 106 μmol/min, T_{gr} = 500°C, p_T = 10 Torr). Extra peaks attributed to other orientations of β-Ga_2O_3 such as (110), ($\bar{8}$01), and (010), are present for the growth with VI/III = 420 only, indicating that the film is polycrystalline.

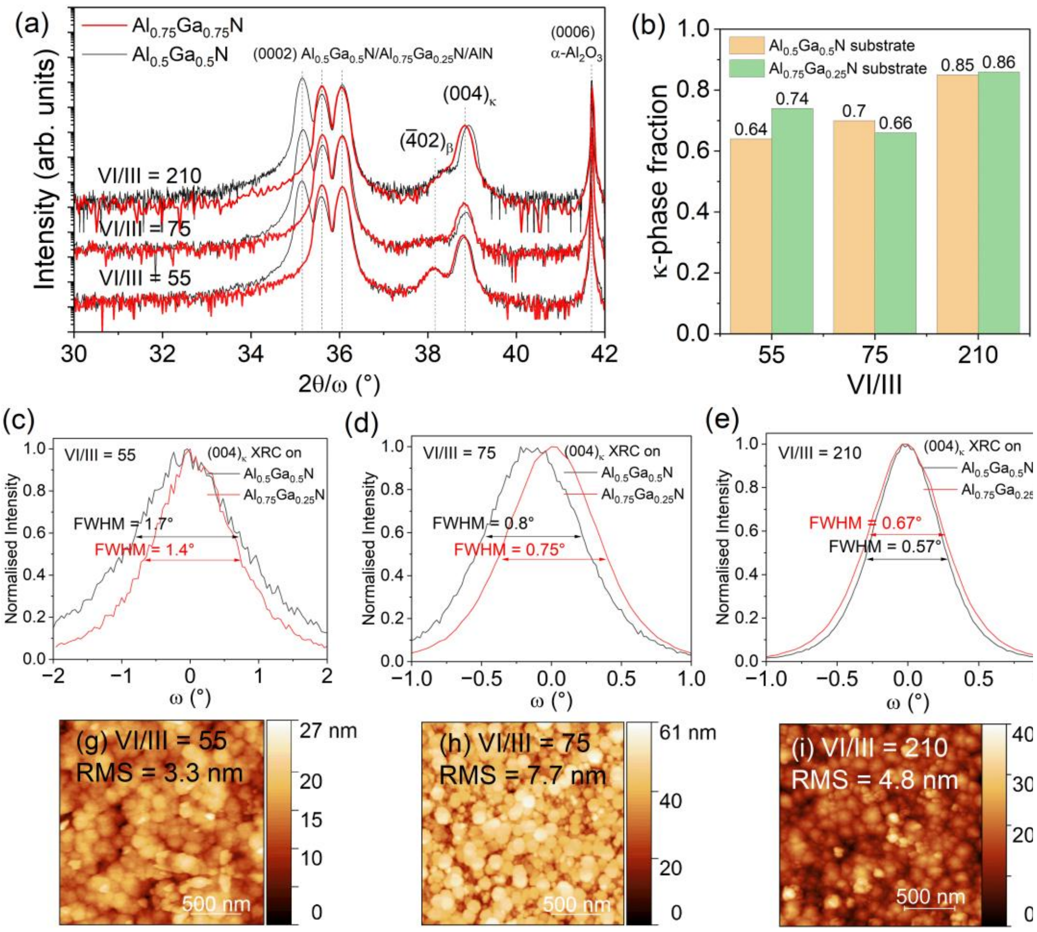

Figure S5: (a) XRD 2θ-ω scans of Ga_2O_3 films grown on $Al_{0.75}Ga_{0.25}N$ (red lines) and $Al_{0.5}Ga_{0.5}N$ (black lines) templates for VI/III = 55, 75, 210 (constant TEG = 106 µmol/min, T_{gr} = 500°C, p_T = 10 Torr), with corresponding $(004)_\kappa$ XRC FWHM shown in (c), (d), and (e), respectively. (b) Comparing the κ-phase fraction of the Ga_2O_3 layer grown on $Al_{0.75}Ga_{0.25}N$ and $Al_{0.5}Ga_{0.5}N$ templates. AFM scans of a 2x2 µm^2 area of Ga_2O_3 grown on $Al_{0.75}Ga_{0.25}N$ for VI/III = (g) 55, (h) 75, (i) 210.

Section B: Structural evolution of Ga_2O_3 film grown under κ-favourable conditions on $Al_xGa_{1-x}N$ on sapphire templates.

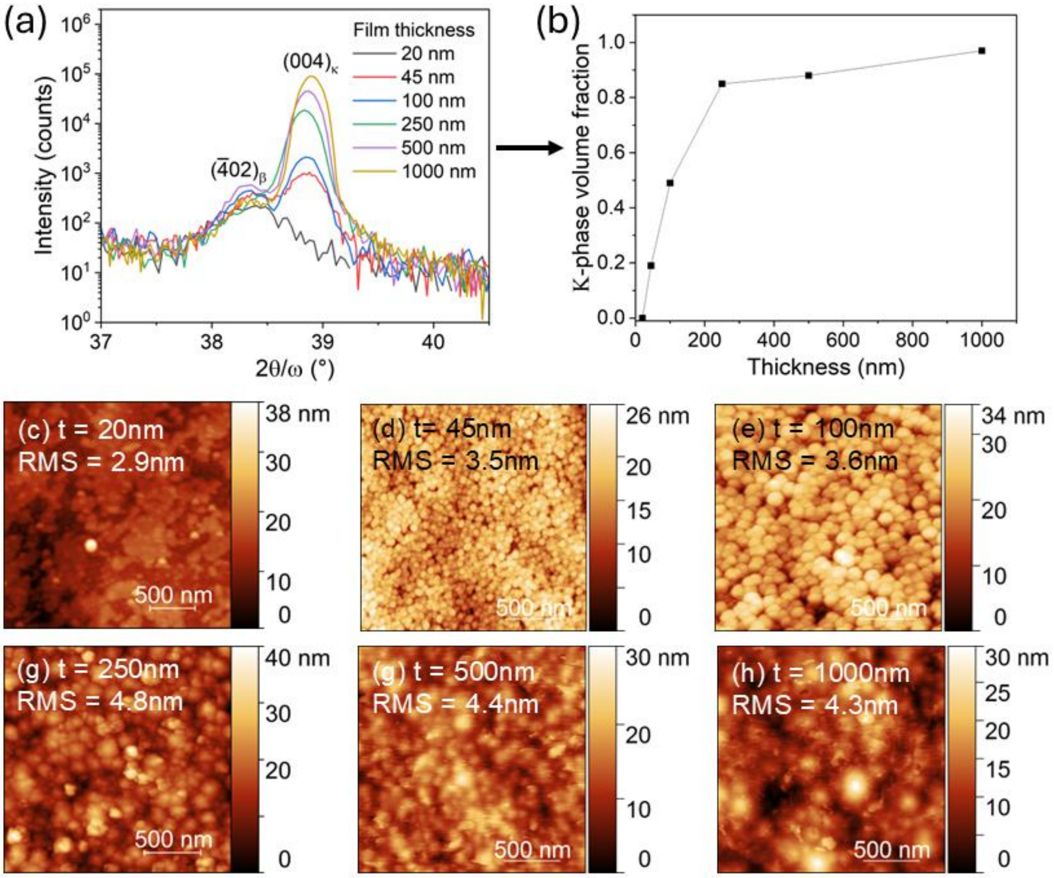

Figure S6: (a) XRD 2θ-ω scans, (b) κ-phase fraction, (c-h) AFM scans of Ga_2O_3 films, 20 – 1000 nm thick, grown on $Al_{0.75}Ga_{0.75}N$ templates, under identical growth conditions (TEG = 106 µmol/min, VI/III = 210, T_{gr} = 500°C, p_T = 10 Torr).

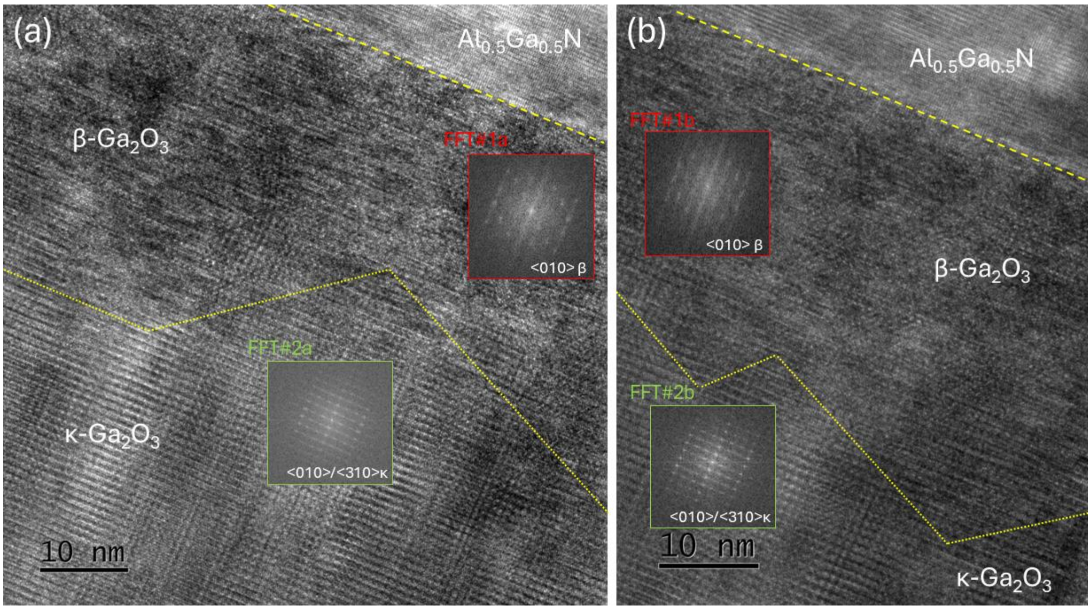

Figure S7: High magnification HR-TEM images of more regions along the film/AlGaN interface of the 250 nm κ-Ga_2O_3 film (KPF ~86%) on $Al_{0.5}Ga_{0.5}N$ template, grown using the conditions: TEG = 106 µmol/min, VI/III = 210, T_{gr} = 500°C, p_T = 10 Torr. Dashed yellow line marks the film/AlGaN interface, and dotted yellow lines mark the approximate boundaries between κ- and β-grains. The phase distribution in regions shown in Fig. S7(a-b) is consistent with the region shown in Fig. 5 of the main manuscript. The β-Ga_2O_3 transition layer appears to be ubiquitous throughout interfacial regions of the sample.
